# Effect of the Sauvé–Kapandji method on the wrist contact surface for distal radial ulnar joint disorders

**DOI:** 10.1186/s12891-024-07652-2

**Published:** 2024-07-11

**Authors:** Tomoaki Suzuki, Daisuke Momma, Nozomu Inoue, Eiji Kondo, Yuichiro Matsui, Norimasa Iwasaki

**Affiliations:** 1 Department of Orthopaedic Surgery, Faculty of Medicine, Graduate School of Medicine, Hokkaido University, Sapporo, Hokkaido Japan; 2 Center for Sports Medicine, Hokkaido University Hospital, Sapporo, Hokkaido Japan; 3 Department of Orthopedic Surgery, Rush University Medical Center, Chicago, USA; 4 Department of General Dentistry, Faculty of Dental Medicine, Hokkaido University, Sapporo, Hokkaido Japan

**Keywords:** Sauvé-Kapandji method, Distal radial ulnar joint disorders, Wrist joint contact area

## Abstract

**Background:**

The Sauvé–Kapandji (S-K) method is a surgical procedure performed for chronic deformities of the distal radial ulnar joint (DRUJ). Changes to the joint contact surface from pre- to postoperatively under physiological in vivo conditions have not yet been determined for this useful treatment. The aim of the present study was therefore to compare the articular contact area of the wrist joint between before and after the S-K method for DRUJ disorders.

**Methods:**

The SK method was performed for 15 patients with DRUJ osteoarthritis and ulnar impaction syndrome. We calculated the Mayo Wrist Score as the patient’s clinical findings and created 3-dimensional bone models of cases in which the S-K method was performed and calculated the contact area and shift in the center of the contact area using customized software.

**Results:**

The Mean modified Mayo Wrist Score improved significantly from 60.3 preoperatively to 80.3 postoperatively (*P* < 0.01). Scaphoid contact area to the radius increased significantly from 112.6 ± 37.0 mm^2^ preoperatively to 127.5 ± 27.8 mm^2^ postoperatively (*P* = 0.03). Lunate contact area to radius-ulna was 121.3 ± 43.3 mm^2^ preoperatively and 112.5 ± 37.6 mm^2^ postoperatively, but this decrease was not significant (*P* = 0.38). Contact area ratio of scaphoid to lunate increased significantly from 1.01 ± 0.4 preoperatively to 1.20 ± 0.3 postoperatively (*P* = 0.02). Postoperative translations of the center of the scaphoid and lunate contact areas were decomposed into ulnar and proximal directions. Ulnar and proximal translation distances of the scaphoid contact area were 0.8 ± 1.7 mm and 0.4 ± 0.6 mm, respectively, and those of the lunate contact area were 1.1 ± 1.7 mm and 0.4 ± 1.1 mm, respectively. This study revealed changes in wrist contact area and center of the contact area before and after the S-K method.

**Conclusion:**

These results may accurately indicate changes in wrist joint contact area from pre- to postoperatively using the S-K method for patients with DRUJ disorder. Evaluation of changes in contact area due to bone surface modeling of the wrist joint using 3DCT images may be useful in considering surgical methods.

**Supplementary Information:**

The online version contains supplementary material available at 10.1186/s12891-024-07652-2.

## Background

Treatment for degeneration of the distal radial ulnar joint (DRUJ) has room for improvement, despite advances in anatomical and biomechanical knowledge of this complex site [1, 2]. Salvage procedures such as Darrach resection [3], hemiresection-interposition arthroplasty [4], and matched resection of the distal aspect of the ulna [5] can generally achieve satisfactory results in elderly patients who do not have high functional demands on the wrist joint. However, the results of these techniques are not always satisfactory for young, active patients who require high functional demands on the wrist and forearm [4, 6–8]. The Sauvé–Kapandji (S-K) method was first described by Sauvé and Kapandji in 1936, combines fixation of the DRUJ with formation of a distal ulnar pseudarthrosis to remedy DRUJ dysfunction. Previous studies have suggested that the S-K method may provide a better treatment option for young, active patients [9–12]. On the other hand, problems with residual ulnar pain and instability have been reported [12–15], and further research is needed on the pathogenesis of these problems. Although many pathological conditions of the DRUJ are considered indications for the S-K method, the effects of surgery for different pathological conditions have yet to be clarified. In particular, the effects of differences in ulnar variance on the joint surfaces before and after surgery remain to be elucidated. Evaluation of joint contact surfaces is considered useful not only in relation to pathological factors, but also in determining treatment strategies. Recently, the use of 3-dimensional (3D) bone models has enabled the evaluation of joint contact surfaces in vivo in three dimensions. The purpose of this study was to evaluate the wrist contact surfaces in vivo before and after the S-K method for DRUJ disorders.

## Methods

### Patients

All patients underwent the S-K method for the treatment of DRUJ disorders between 2010 and 2022. Computed tomography (CT), plain X-rays, and clinical assessment data were collected pre- and postoperatively. CT was imaged in neutral position. The modified Mayo Wrist Scoring system was used for pre- and postoperative assessments of pain and wrist range of motion.

### Surgical technique

The distal ulna was incised dorsally, the fifth extensor compartment was opened, the extensor digit minimi was retracted, and the articular capsule was incised. The DRUJ was decorticated and ulnar osteotomy was performed to create a 1-cm pseudoarthrosis proximal to the ulnar metaphysis. One or two 3.5-mm cannulated screws were placed across the DRUJ. A bone graft was placed from the resected ulna and filled into the arthrodesis site. The proximal ulnar osteotomy was stabilized by passing the tendon of the severed extensor carpi ulnaris muscle through a 2.7-mm drilled bony hole and interracing suture to the tendon again. Postoperatively, a long arm splint was applied for 2 weeks, followed by initiation of active and passive motions of the wrist.

### Creation of 3D bone models

CT was performed using a 320-slice multidetector 3D scanner with wide field-of-view (FOV) (Aquilion One; Canon Medical Systems, Tochigi, Japan). Settings were: slice thickness, 0.5 mm; slice interval, 0.5 mm; matrix, 512 × 512; and FOV, φ500 mm. During image acquisition, the forearm was kept in the mid-arm position. CT images of the wrist were obtained preoperatively and within 3 months postoperatively. CT images of each wrist joint were imported in DICOM format and segmented using segmentation software (Mimics 21R; Materialise, Leuven, Belgium). Three-dimensional images of the radius, ulna, lunate and scaphoid were reconstructed and exported as point cloud and polygon models using the same software package. The 3D models of the radius, ulna, scaphoid, and lunate were analyzed using custom-written software created in Microsoft Visual C + + in the Microsoft Foundation Class programming environment (Microsoft, Redmond, WA) [16–18].

**Fig. 1:**
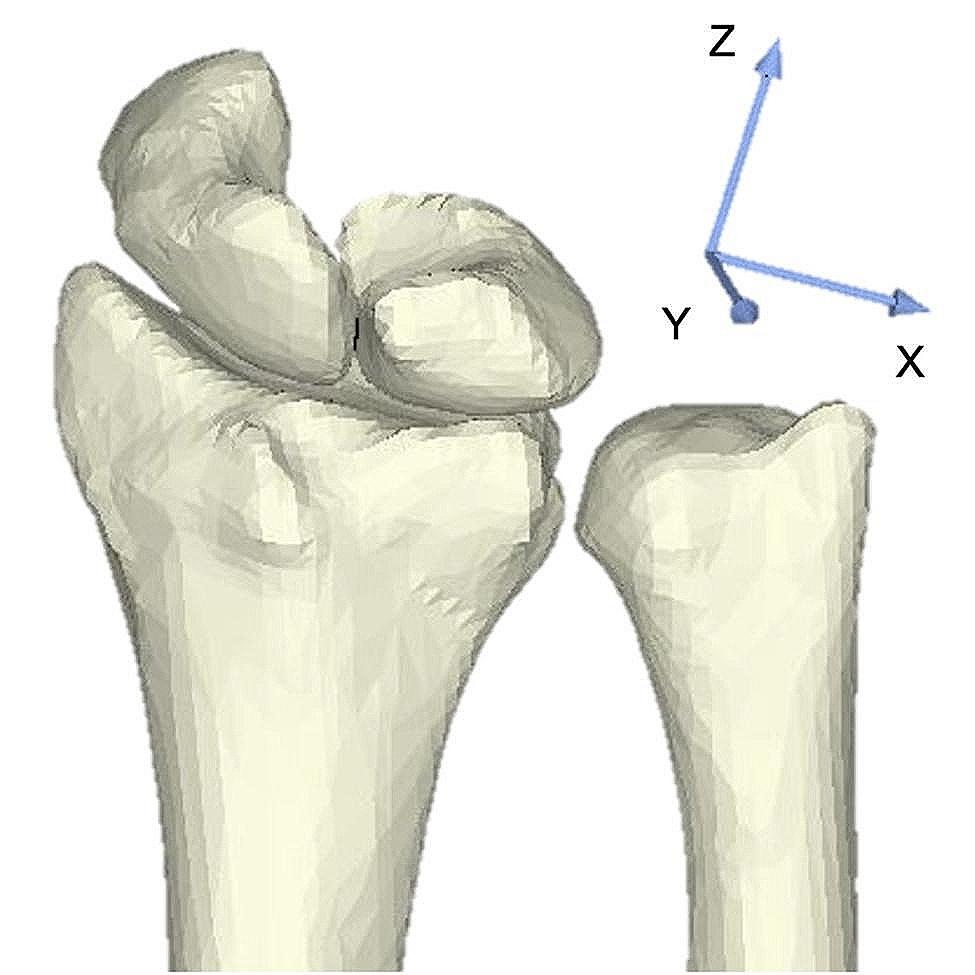
Anatomical coordinate system of the wrist. Translations along the X-, Y-, and Z-axes indicate the radial (+)/ulnar (−), dorsal (+)/volar (−), and distal (+)/proximal (–) directions, respectively.

Least-distance distributions between surfaces for the radius and scaphoid models, radius and lunate models, and ulna and lunate models were calculated from a point-to-point distance calculation algorithm using custom-written software [19, 20]. Articular contact areas were defined as those areas where least distances were below a certain threshold. Distance thresholds were determined with reference to previous studies on distances in the wrist articular space [21, 22]. The distance threshold was set at 2.0 mm for each of the radiocarpal and ulnocarpal joints. Contact area of the scaphoid to the radius and the lunate to the radius-ulna were calculated from the bone models using custom-written software. In addition, contact area ratio of scaphoid to lunate was calculated by dividing the lunate contact area from the scaphoid contact area. The center of the area where the scaphoid and lunate contacted the radius-ulna was calculated, and the shift from preoperatively to postoperatively was calculated using custom-written software. The postoperative immobilization of the DRUJ made the radius difficult to separate from the ulna, so calculations used the articular surface of the radius-ulna preoperatively and the combined articular surface of the radius-ulna, including the grafted bone postoperatively. When measuring the contact area and center of the contact area, the scaphoid and lunate were each combined with the radius-ulna, respectively. A validated 3D–3D registration method was used to evaluate translation of the center of the contact area, and a preoperative–postoperative transformation matrix was obtained [23, 24](Supplementary Table 1). The International Society of Biomechanics standard anatomical coordinate system for the wrist was used (Fig. 1) [25].

### Statistical analyses

Pre- and postoperative modified Mayo Wrist Scores and contact area were statistically compared using a paired *t*-test, with values of *P* < 0.05 considered significant. Data are presented as mean ± SD and corresponding 95% confidence intervals.

## Results

The S-K method was performed for 15 patients with DRUJ osteoarthritis and ulnar impaction syndrome. There were 6 male and 9 female patients whose mean age was 63.4 years (range, 45–81 years). Six patients with DRUJ OA required additional tendon reconstruction. All patients were rated “Excellent” or “Good” on the modified Mayo Wrist Score system at 5–7 months postoperatively. This score improved significantly from 60.3 ± 7.6 preoperatively to 80.3 ± 6.9 postoperatively (Table 1). Using the technique described by Gelberman et al., mean preoperative ulnar variance on posteroanterior X-rays was 3.4 mm ulnar plus variance (range, 0–10 mm ulnar plus variance). Ten cases showed ulna plus variance (mean, 5.1 mm; range, 2.5–10 mm) and 5 cases showed ulna non-plus variance (0 mm in all cases). The postoperative value approached 0 mm ulnar variance (range, 0 mm ulnar variance to 1 mm ulnar negative variance) (Table 1).

**Table 1:** Participant characteristics

Case	**Affected hand**	Diagnosis	Ulnar variacne		**Mayo wrist score**	
			Pre- operation	Post- operation	Pre- operation	Post- operation
1	L	Ulnar impaction syndrome	3.5	0	55	85
2	L	DRUJ OA	3.0	0	55	80
3	R	DRUJ OA	4.0	0	55	70
4	R	DRUJ OA	10	0	70	90
5	R	Ulnar impaction syndrome	10	0	70	70
6	R	Ulnar impaction syndrome	2.5	-1	75	90
7	R	DRUJ OA	2.5	0	70	85
8	R	DRUJ OA	6.0	0	60	85
9	R	Ulnar impaction syndrome	6.0	0	60	80
10	L	Ulnar impaction syndrome	3.5	0	50	80
11	R	DRUJ OA	0	0	65	80
12	L	DRUJ OA	0	0	55	70
13	R	DRUJ OA	0	0	50	70
14	L	DRUJ OA	0	0	55	75
15	R	DRUJ OA	0	0	55	90
Mean			3.4	-0.1	60.3	80.3
P value per- vs post-operation			< .001		< .001	

**Fig. 2:**
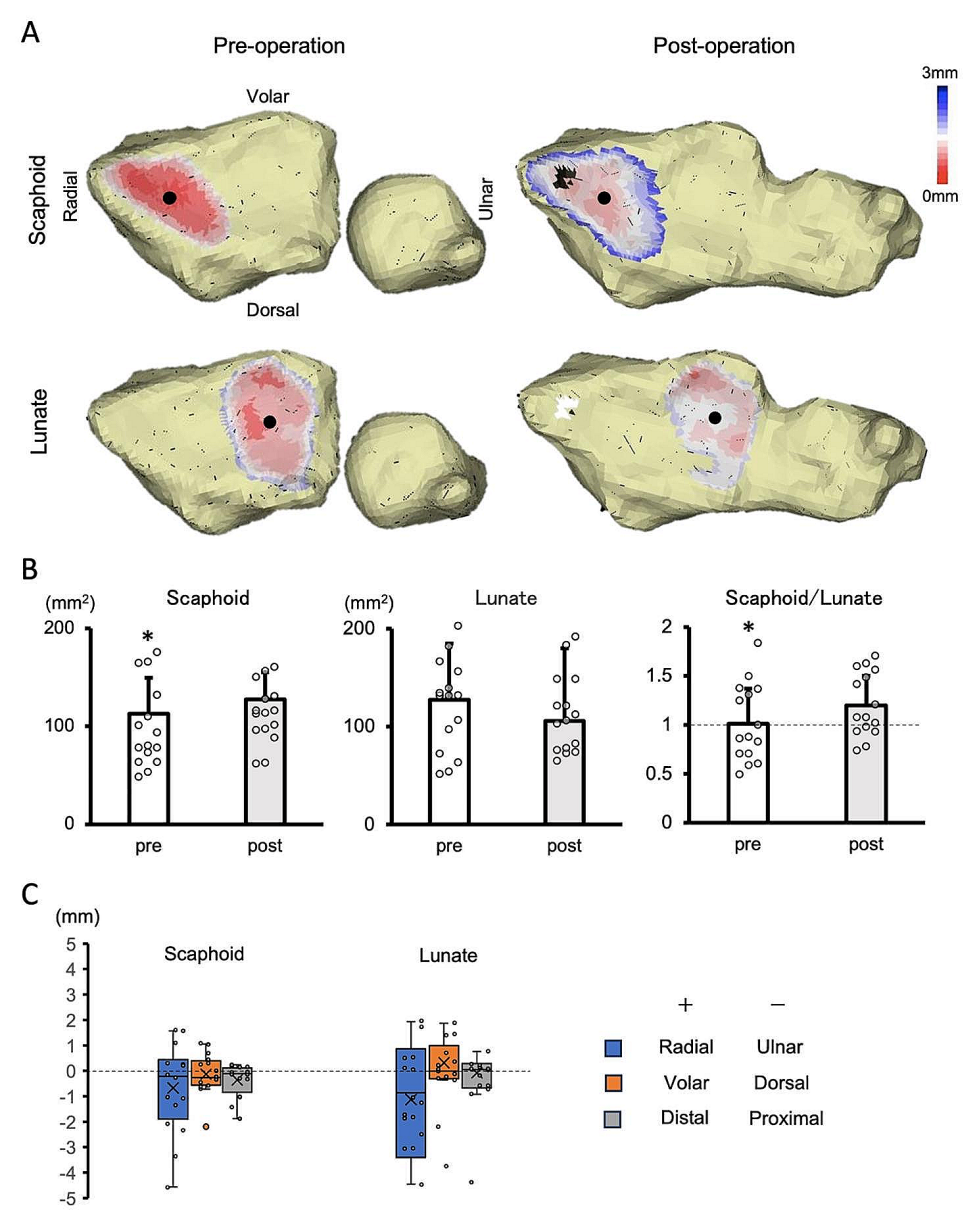
(**A**) The representative bone models and joint contact area. Left is preoperative and right is postoperative wrist joint contact areas. Upper is the area where the scaphoid and lower is the area where the lunate contacts the articular surface. Wide and narrow joint spaces are indicated by blue and red areas, respectively. The black dots indicate the center of the joint contact area. (**B**) Values of joint surface contact area of scaphoid and lunate and their area ratios. *P < 0.05, paired *t-*test. (**C**) The postoperative translation of the center of the radioscaphoid and radiolunate contact area. *N* = 15/group

Scaphoid contact area increased significantly from 112.6 ± 37.0 mm^2^ preoperatively to 127.5 ± 27.8 mm^2^ postoperatively (*P* = 0.03). Lunate contact area was 121.3 ± 43.3 mm^2^ preoperatively and 112.5 ± 37.6 mm^2^ postoperatively, but this decrease was not significant (*P* = 0.38). On the other hand, the contact area ratio of scaphoid to lunate increased significantly from 1.01 ± 0.4 preoperatively to 1.20 ± 0.3 postoperatively(*P* = 0.02) (Fig. 2A, B).

The postoperative translation of the center of the scaphoid and lunate contact areas was decomposed into ulnar and proximal directions. Ulnar and proximal translations of the scaphoid contact area were 0.8 ± 1.7 mm and 0.4 ± 0.6 mm, respectively, and those of the lunate contact area were 1.1 ± 1.7 mm and 0.4 ± 1.1 mm, respectively. Almost no translation was seen in the dorsal palmar direction (0 ± 0.6 mm and 0.2 ± 1.3 mm, respectively) (Fig. 2A, C).

**Fig. 3:**
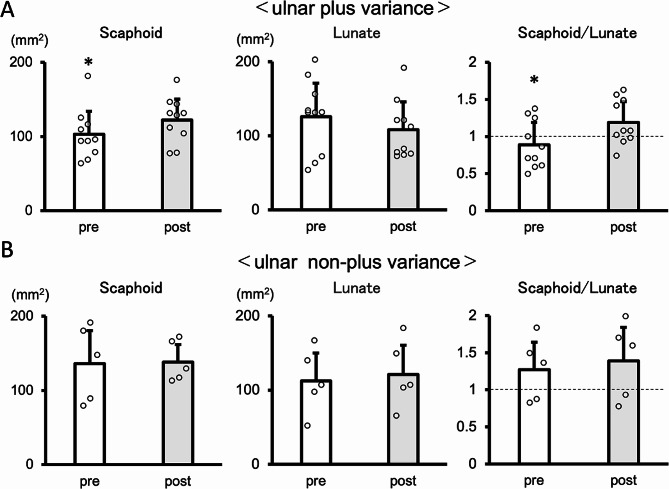
Values of joint surface contact area of scaphoid and lunate and their area ratios. (**A**) ulnar plus variance(*N* = 10/group), (**B**) ulnar non-plus variance (*N* = 5/group *P < 0.05, student’s test

Contact area was then compared between cases with ulna plus variance and non-plus variance. Scaphoid contact area in plus cases increased significantly from 102.9 ± 31.0 mm^2^ preoperatively to 122.2 ± 28.2 mm^2^ postoperatively (*P* = 0.01). Lunate contact area tended to decrease from 125.7 ± 45.3 mm^2^ preoperatively to 108.2 ± 36.0 mm^2^ postoperatively, but this change was not significant (*P* = 0.22). On the other hand, scaphoid contact area in non-plus cases was similar, at 136.1 ± 44.3 mm^2^ preoperatively and 138.2 ± 23.9 mm^2^ postoperatively (*P* = 0.89). Lunate contact area tended to increase from 112.4 ± 37.6 mm^2^ preoperatively to 121.1 ± 39.2 mm^2^ postoperatively, but this change was not significant (*P* = 0.42) (Fig. 3).

## Discussion

This study offers the first in vivo evaluation of wrist contact areas before and after the S-K method for DRUJ disorders. The present study demonstrated that contact areas of the wrist joint changed between before and after the S-K method. After the S-K method, the center of the contact area of the radius and ulna moved ulnarly. These results indicate that the S-K method has potential to realign carpal alignment that has deviated radially due to DRUJ disorder toward the normal position.

Although several studies have been conducted to evaluate joint contact area, none have investigated changes in joint contact area associated with DRUJ disorders and their treatment. This study therefore used an in vivo CT bone model analysis system to clarify changes in joint contact area after the S-K method for DRUJ disorders. Contact areas of the scaphoid and lunate to radius-ulna were calculated before and after the S-K method using in vivo 3D methods.

A previous study on contact areas of the wrist joints used cadaveric wrist joints to show that overall scaphoid contact area was 1.47 times greater than that of the lunate [26]. In another report, force transmittion to the radio-ulnocarpal joint is 55% for the radioscaphoid joint and 35% for the radiolunate joint [27], indicating that the wrist joint transmits more load to the scaphoid. In the present study, scaphoid contact area was significantly increased and lunate contact area tended to decrease, increasing the contact area ratio of scaphoid to lunate from 1.01 ± 0.4 to 1.20 ± 0.3. This result indicates that the S-K method approached the stress distribution of the normal wrist joint.

Cadaveric experiments have reported that in a wrist joint with neutral ulnar variance, 82% of stress is transferred to the radiocarpal joint and 18% to the ulnocarpal joint; the more ulnar variance increases, the greater the stress on the ulnocarpal joint [1, 28]. The centers of the scaphoid and lunate contact areas were shifted ulnarly after the S-K method. The pressure on the scaphoid and lunate caused by instability of the DRUJ and ulnar thrust was found to be reduced by the S-K method. In the present study, when the S-K method was performed in patients with ulnar plus variance, the contact area was significantly changed compared to patients with ulnar non-plus variance. However, interestingly, rates of improvement in Mayo Wrist score were comparable. These results indicated that relief of symptoms was influenced by S-K method itself, that is stabilizing DRUJ and decompression of ulnolunate joint, and was not influenced by the change of scaphoid and lunate contact areas. However, changing the contact area in the “normal direction” may prevent future occurrence of OA.

Several limitations to this study need to be kept in mind. First, the measurement technique used a 3D model of the bone surface. Second, actual wrist joint stresses were not measured. Third, significant differences between pre- and postoperative joint contact areas may not always be present, as results vary widely from patient to patient. In addition, the present study could not identify any direct relationship between the pathology of the wrist joint and the causal relationship between treatment efficacies. However, these results may accurately indicate changes in wrist joint contact area from pre- to postoperatively using the S-K method for patients with DRUJ disorder.

## Conclusions

It was suggested that the S-K method may improve symptoms by stabilizing the DRUJ as well as altering radial-ulnar articular surface contact of the scaphoid and lunate. Evaluation of changes in contact area before and after S-K method due to bone surface modeling of the wrist joint using 3DCT images may be useful in considering the indications for this method.

### Electronic supplementary material

Below is the link to the electronic supplementary material.

Supplementary Material 1

## Data Availability

All data generated or analyzed during this study are included in this published article and its supplementary information files.

## Abbreviations

S-K: Sauvé–Kapandji
DRUJ: Distal radial ulnar joint
3D: 3-dimensional
CT: Computed tomography
FOV: Field-of-view
